# Co-infection of Neonatal Plasmodium knowlesi Malaria With Congenital Cytomegalovirus: The First Reported Case From Bahrain

**DOI:** 10.7759/cureus.100574

**Published:** 2026-01-01

**Authors:** Abdulrahman D Mohroofi, Hadhami Ben Turkia, Fatema Aljowder, Dana Althawadi, Ramaning Loni

**Affiliations:** 1 Medicine, Arabian Gulf University, Manama, BHR; 2 Pediatrics, King Hamad University Hospital, Manama, BHR; 3 Pathology, King Hamad University Hospital, Manama, BHR; 4 Pediatric Emergency, King Hamad University Hospital, Manama, BHR; 5 Pediatric Intensive Care Unit, King Hamad University Hospital, Manama, BHR

**Keywords:** bahrain, coinfection, cytomegalovirus, imported infection, neonatal malaria, plasmodium knowlesi

## Abstract

Neonatal malaria is rare due to protective maternal immunity and breastfeeding, particularly in non-endemic areas of the globe. *Plasmodium (P.) knowlesi *is a zoonotic species mainly found in Southeast Asia, and very few cases have been reported worldwide in the pediatric population. We report the first documented case of congenital malaria in Bahrain due to *Plasmodium knowlesi*.

A 26-day-old, Pakistani male infant presented with a one-day history of fever, jaundice, lethargy, and hepatosplenomegaly. Laboratory investigations revealed pancytopenia and elevated inflammatory markers. Despite negative rapid testing for malaria antigen, the peripheral blood smear confirmed the presence of *P. knowlesi.* The mother’s malaria PCR and smear were negative. The infant also tested positive for cytomegalovirus with high blood and urine viral loads. He was treated with intravenous artesunate and ganciclovir, followed by oral valganciclovir.

The case spotlights the importance of considering congenital malaria in neonates with nonspecific systemic symptoms, even in non-endemic areas. Blood smear remains essential for the diagnosis even when rapid tests are negative. Co-infection with malaria may occur, and it may complicate the clinical picture. Clinicians in malaria-free regions must be aware of imported infections due to rising global migration.

## Introduction

Neonatal malaria is considered an extremely rare infection given the protective effect of maternal immunity after birth [1]. It is difficult to differentiate between congenital malaria (CM) and acquired neonatal malaria (NM) when symptoms manifest in the first month of life [2]. Congenital malaria is defined as an asexual parasitic protozoan detected in cord blood or peripheral blood in the first week of life, as maternal erythrocytes containing parasites can cross the placental barrier and infect neonatal red blood cells [1,2]. The prevalence of congenital malaria varies depending on the endemicity of the infection, with an overall prevalence of 6.9% (95% CI: 4.8-7.9%), which can reach up to 46.7% in endemic countries like Nigeria [3]. On the other hand, acquired neonatal malaria is transmitted by an Anopheles mosquito bite after birth within the first 28 days of life [4]. Malaria in newborns can be caused by one of five *Plasmodium (P.)* species: *P. vivax, P. falciparum, P. ovale, P. malariae,* and *P. knowlesi* [5]. Statistically, *P. knowlesi *is a less common infection compared to *P. falciparum* and *P. vivax* [6]. Malaysia, Brunei, and Indonesia are considered among the countries with the highest rate of *P. knowlesi* infection, which has been associated with the geographical presence of the natural hosts and vectors for the infection, specifically monkeys (Macaca fascicularis) [7].

Worldwide, congenital cytomegalovirus (CMV) infection is considered the most widespread congenital infection, with an estimated prevalence ranging between 0.1% and 2% of all infected pregnancies [8]. Congenital CMV infection occurs through transplacental transmission, and among infected babies, only around 10% will have symptoms while the majority are asymptomatic at birth [8,9]. Both neonatal malaria and congenital CMV infection can present with general nonspecific symptoms [9,10].

Malaria infection in pregnancy has been hypothesized to increase intrauterine transmission of CMV and vice versa, with no exact pathophysiology [8].

We report the first case of co-infection of neonatal malaria with congenital CMV in a tertiary hospital in the Kingdom of Bahrain.

## Case presentation

A 26-day-old Pakistani boy presented to the Emergency Department (ED) with a history of documented fever for one day associated with yellowish discoloration of the eyes and face. The baby was born via spontaneous vaginal delivery at 36 weeks of gestation with a birth weight of 2107 grams, length of 47 cm, and head circumference measuring 31 cm. His Apgar scores were 9 and 10 at 1 and 5 minutes, respectively. He was admitted to the Neonatal Intensive Care Unit (NICU) for jaundice and suspected sepsis. The septic workup came back negative, and the patient was discharged on day 5 of life. He passed the hearing screening bilaterally, and his newborn screening was negative. Since discharge from the NICU, the infant has been doing well at home, is breastfed exclusively, and is thriving well. The mother was an 18-year-old primigravida. She migrated from Pakistan to Bahrain during the first trimester. She was diagnosed with gestational diabetes mellitus and controlled on a diabetic diet. Rubella, Syphilis, Hepatitis B, and human immunodeficiency virus (HIV) serologies were negative, and antenatal fetal scans were normal. The mother did not experience any symptoms during pregnancy.

Upon arrival at the ED, the baby was vitally stable and afebrile. He was sick-looking, hypoactive, and jaundiced. Further examination revealed hepatosplenomegaly and hypotonia; the primitive reflexes were intact otherwise. There were no bruises or sites of active bleeding.

Initial investigations showed leukopenia, thrombocytopenia, high inflammatory markers, and increased gamma-glutamyltransferase enzyme (Table 1).

**Table 1:** Initial laboratory investigations

Investigation	Result	Reference Range
White cell count	4*109/L	(5 - 16 *109/L)
Absolute neutrophil count	1.3*109/L	(1.1 – 6.6 *109/L)
Hemoglobin	12.5 g/dl	(10 – 14 g/dl)
Platelets	34*109/L	(150 – 450 *109/L)
C-reactive Protein	202 mg/l	(0 – 10 mg/l)
Procalcitonin	25 ng/ml	(< 0.05 ng/mL)
Gamma-glutamyl transferase	280 U/I	(0 – 185 u/l)
Lactate dehydrogenase (LDH)	373 u/L	(120 – 300 U/L)
D-dimer	6 mg/L	(< 0.5 mg/L)
Total bilirubin	147 umol/l	5 – 20 umol/l
Direct bilirubin	28 umol/l	<5 umol/l

In view of the organomegaly and bicytopenia, other investigations were requested, including peripheral blood smear, reticulocytes, lactate dehydrogenase (LDH), stool occult blood, D-dimer, TORCH (Toxoplasmosis, Rubella, Cytomegalovirus, Herpes, and Other agents) serology, and hemoglobin electrophoresis. Full septic workup, including blood, cerebrospinal fluid, and urine cultures, was collected, and the patient was commenced on cefotaxime and ampicillin.

On day 2 of admission, the baby’s clinical condition deteriorated, and his labs showed a drop in hemoglobin from 12 g/dl to 7 g/dl. The patient was transferred to the Pediatric Intensive Care Unit (PICU), where blood and platelet transfusions were given. All cultures turned out to be negative. Other tests showed high LDH and D-dimer, positive stool occult blood, negative human immunodeficiency virus (HIV) polymerase chain reaction (PCR), parvovirus B19 PCR, and negative TORCH screening for Rubella, Toxoplasma, Herpes, and Syphilis. Interestingly, lab results reported positive immunoglobulin M antibodies of cytomegalovirus with a significant viral load in blood and urine (5,940,480 copies/mL and 3360 copies/mL, respectively).

**Figure 1:**
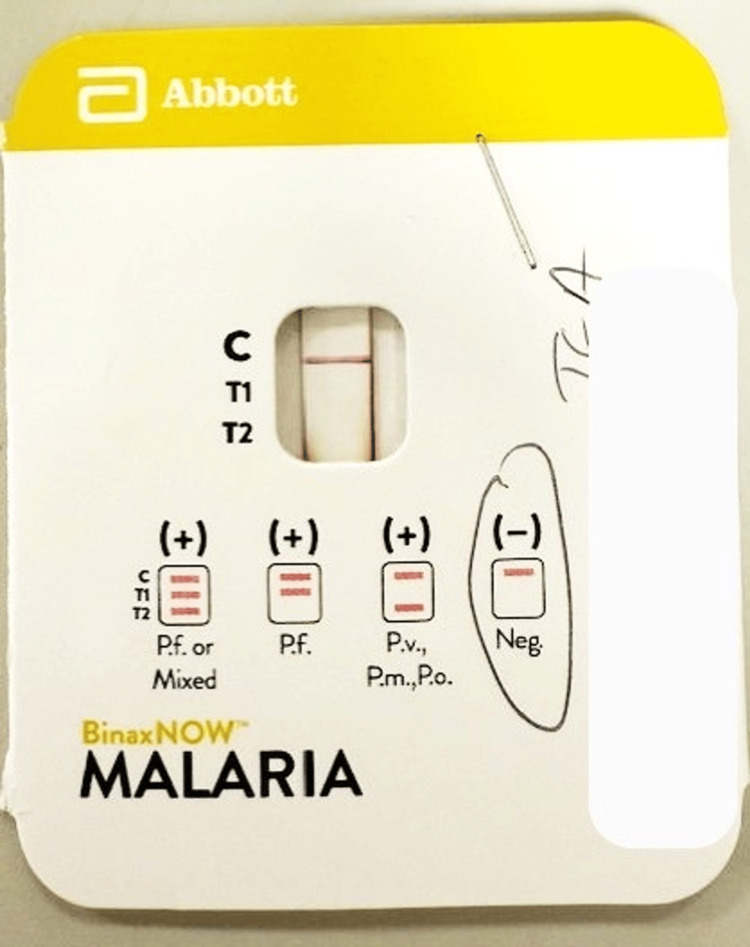
Rapid BinaxNOW malaria antigen test of the patient (negative)

The malaria antigen test was negative for four *Plasmodiu*m species (*falciparum, vivax, ovale, and malaria*) (Figure 1).

**Figure 2:**
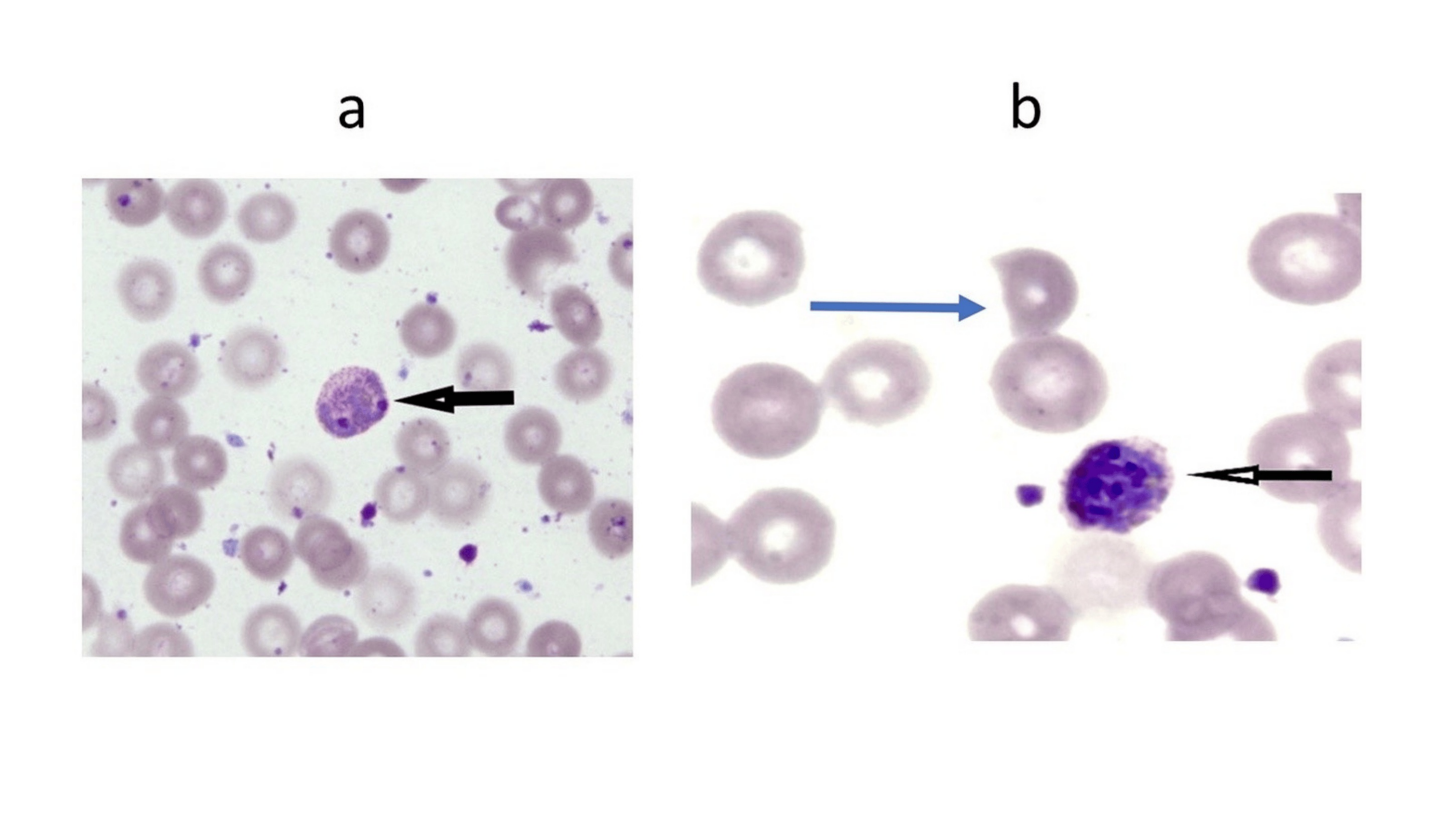
a: Early trophozoite stage of Plasmodium knowlesi, appearing as ring forms with double chromatin dots and found in pairs or triplets within a single erythrocyte (black arrow). b: Schizont stage of Plasmodium knowlesi contains up to 16 merozoites (black arrow). Schistocytes/RBC fragment (blue arrow) is seen with a parasitemia of 0.6%.

Peripheral blood smear reported pancytopenia with the presence of protozoal parasites suggestive of *Plasmodium knowlesi* by the detection of the trophozoite and schizont stages (Figure 2).

**Figure 3:**
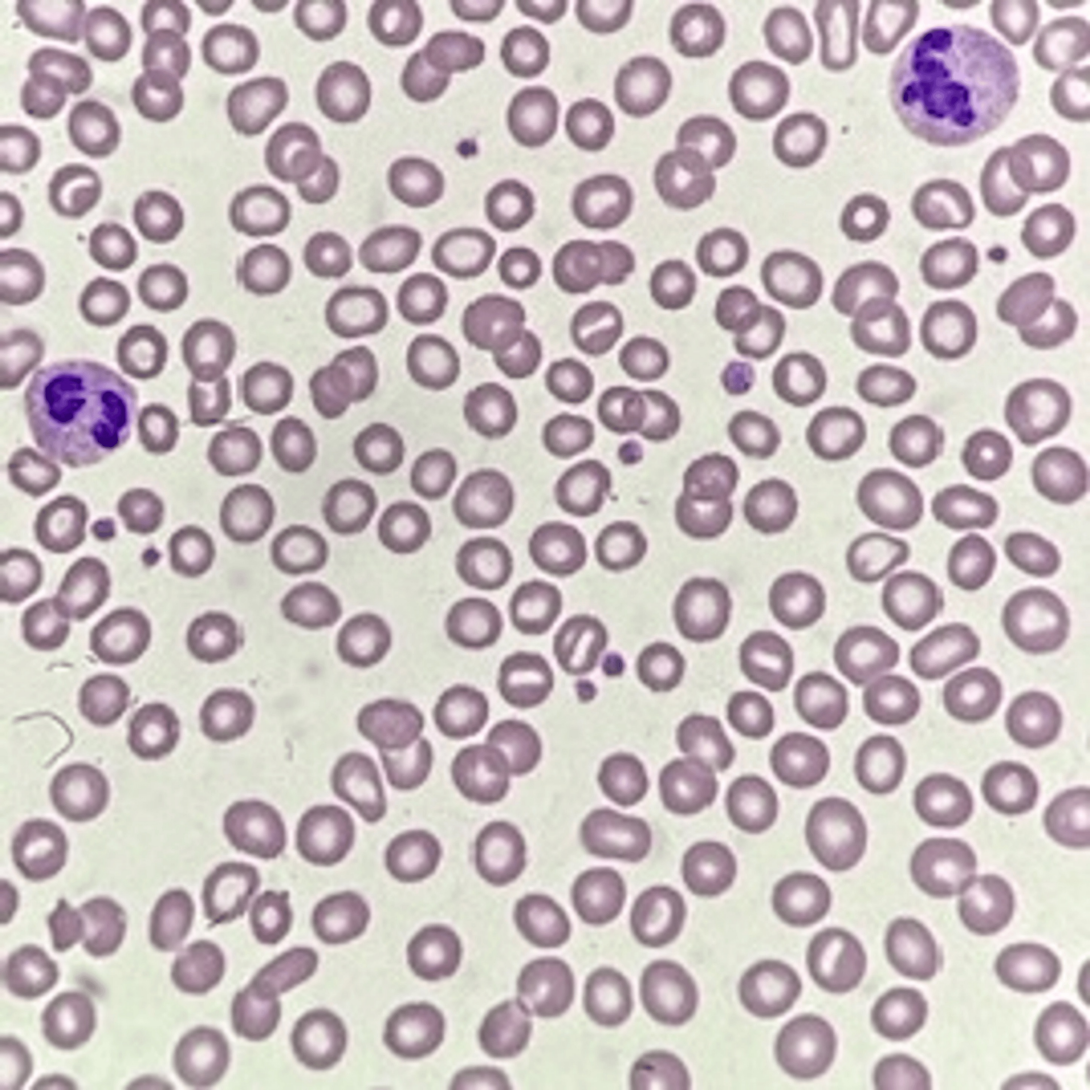
Mother’s peripheral smear showing microcytic hypochromic anemia. No malaria parasite was detected.

The infant underwent a multisystem assessment for malaria and CMV complications, including echocardiography, brain ultrasound, and ophthalmologic and hearing assessment; all were normal. The mother was tested for malaria antigen and peripheral smear, which both came back negative (Figure 3).

**Figure 4:**
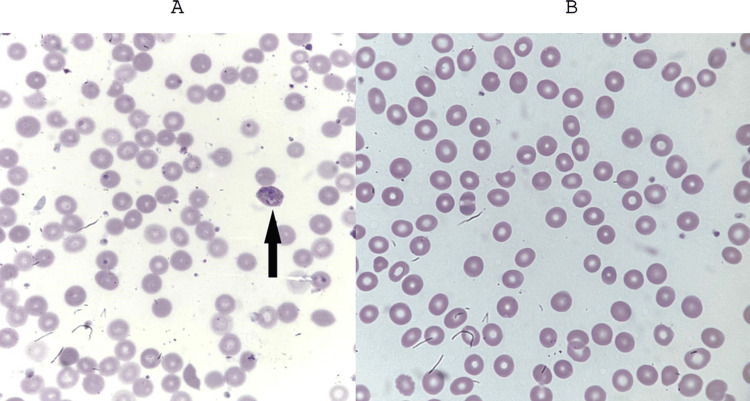
A: peripheral smear of the infant pointing to a trophozoite stage of Plasmodium knowlesi (black arrow), with a parasitemia of 0.2%; B: peripheral smear on day 11 with eradication of parasite (parasitemia 0%)

The baby was started on intravenous artesunate 3 mg/kg/day and gancyclovir 6 mg/kg, both for seven days. The patient improved clinically dramatically; he did not require further blood products. Parasitemia was monitored on a daily basis until eradication on day 11, and his complete blood count normalized on day 13 (Figure 4).

CMV viral load was followed up during his admission, which reduced to 3360 copies/mL, and then to undetectable. On the discharge day, the infant’s lab values showed total bilirubin of 18 umol/l, hemoglobin of 9.4 g/dl, and platelets of 360*10^9/l. The baby was then discharged on oral valganciclovir 16 mg/kg/dose twice daily for a 6-month duration with close follow-up and monitoring of drug side effects, development, hearing, and vision.

## Discussion

Occurrence of neonatal malaria is rare because of several protective factors, and detection of the disease could be challenging if the rapid antigen detection test is negative. Maternal immunoglobulin G antibodies, lactoferrin, and immunoglobulin A found in breast milk and fetal hemoglobin all play a role in inhibiting the growth of parasites and providing protection to neonates from any infection in the first few months of life [11].

Among the 17 Middle Eastern countries, 6 (Bahrain, Qatar, Kuwait, UAE, Oman, and Saudi Arabia) are located in the Arabian Peninsula and are part of the Gulf Cooperation Council (GCC). All GCC countries, with the exception of certain regions in Saudi Arabia and Oman, are free from indigenous malaria [12]. The World Health Organization declared Bahrain malaria-free in 2012. Still, imported malaria cases were reported by the Bahrain Ministry of Health, mainly because of the large expatriate workforce coming from malaria-endemic countries such as Pakistan, India, and Bangladesh [13]. One thousand five hundred and seventy-two infected patients were reported in Bahrain between 1992 and 2001; all of them were imported cases. Furthermore, only Yemen has reported 33 cases that were diagnosed in children [13].

According to a study done in Malaysia, 9% of the pediatric malaria cases were caused by *P. knowlesi* as compared to 41% and 32% caused by *P. vivax* and *P. falciparum, *respectively [6]. Only two studies in the region have reported a *P. Knowlesi *malaria infection among children [14]. To our best knowledge, this is the first pediatric malaria caused by *P. knowlesi* in GCC countries.

Interestingly, the prevalence of congenital CMV infection was higher among infants born with maternal placental malaria parasitaemia. Simultaneously, the predisposition of the placenta to become infected with malaria is higher after a CMV infection [8]. Chandelia S et al. reported a case of congenital CMV infection but with *P. vivax *malaria in an immunocompetent neonate [15]. It has been reported that primigravida mothers possess a higher risk of being infected with malaria and transmitting the infection to their fetuses compared to multigravida [16].

Both neonatal malaria and congenital CMV infections can present with fever, jaundice, reduced feeding, anemia, and/or hepatosplenomegaly similar to our patient [9,10]. According to the Barber et al. study, all children infected by *P. knowlesi *presented with anemia and thrombocytopenia, which was the case in our patient [12]. However, a case series done in Ethiopia reported that neonatal malaria can present atypically in the form of absence of fever, respiratory distress, and abdominal distention along with the other general signs and symptoms. Additionally, patients can present late, which could be attributed to the temporary immunity from maternal IgG antibodies transferring to the fetus [17,18].

The gold standard diagnostic test for neonatal malaria is a Giemsa-stained peripheral blood smear [11]. Even though congenital malaria is transmitted vertically, the maternal blood film is usually negative. This is thought to be attributed to the clearance of malaria from maternal and placental blood at the time of investigation [2,19]. Likewise, in our case, the mother’s blood smear was negative. On the other hand, measurement of CMV PCR viral load in either urine or saliva is shown to be reliable for the diagnosis of congenital CMV in newborns [20].

As a single case study, there were a few limitations. The inability to process CMV PCR on dried blood spot collected for newborn screening led to difficulty in the confirmation of congenital CMV rather than a post-natal infection.

## Conclusions

This case highlights the critical importance of maintaining a high index of suspicion for congenital infections, such as malaria and cytomegalovirus (CMV), in neonates presenting with nonspecific systemic symptoms, even in regions certified as malaria-free. The coexistence of *Plasmodium knowlesi* malaria and congenital CMV in this infant underscores the potential for dual infections to obscure clinical diagnosis and exacerbate disease severity. It further illustrates that malaria rapid diagnostic tests may yield false-negative results, reinforcing the essential role of peripheral blood smear examination in confirming infection, particularly for uncommon species such as *P. knowlesi*.

Given the growing population mobility and global migration from malaria-endemic regions, clinicians should remain vigilant for imported infections and consider concurrent viral etiologies like CMV that may influence immune response, hematologic profiles, and overall neonatal outcomes.
